# In Vitro Antioxidant Properties of Mitratonic Remedy in Tea Form and In Vivo Protection Against Methanol-Induced Oxidative Stress in Wistar Rats

**DOI:** 10.1155/sci5/9944914

**Published:** 2025-08-18

**Authors:** Acharaporn Issuriya, Sineenart Sanpinit, Surasak Limsuwan, Jo Aan Goon, Palika Wetchakul

**Affiliations:** ^1^Division of Health and Applied Sciences, Faculty of Science, Prince of Songkla University, Hat Yai, Songkhla 90110, Thailand; ^2^Division of Applied Thai Traditional Medicine, School of Medicine, Walailak University, Thasala, Nakhon Si Thammarat 80160, Thailand; ^3^Center of Excellence in Tropical Pathobiology, Walailak University, Thasala, Nakhon Si Thammarat 80160, Thailand; ^4^Faculty of Traditional Thai Medicine, Prince of Songkla University, Hat Yai, Songkhla 90110, Thailand; ^5^Department of Biochemistry, Faculty of Medicine, Universiti Kebangsaan Malaysia, Cheras, Kuala Lumpur 56000, Malaysia

**Keywords:** animal model, folk medicine, Mitra tea, *Mitragyna speciosa*, Thai remedy

## Abstract

Natural healthcare products with antiaging properties are attracting growing interest, and Thailand has a rich tradition of using herbal remedies for disease treatment and overall well-being. Mitra tea, derived from the traditional Mitratonic Remedy, is a Thai herbal formulation that has been used by local traditional healers for over 20 years and is officially recognized by the Department of Thai Traditional and Alternative Medicine, Ministry of Public Health, Thailand. Traditionally, it has been used to promote vitality, strengthen the body, and support longevity. However, scientific evidence supporting these traditional claims remains limited. Therefore, this study aimed to investigate the potential of Mitratonic Remedy in the form of herbal tea, herein referred to as Mitra tea. This study evaluated the antioxidant activity of Mitra tea through various antioxidant in vitro assays and analysis, alongside an in vivo male Wistar rats model with induced oxidative stress. Results demonstrated remarkably strong antioxidant properties, with notable DPPH scavenging activity (0.40 mg/mL) and high ferric-reducing antioxidant power (FRAP: 2551.95 mM FeSO_4_ equivalent/mg sample). Additionally, Mitra tea exhibited exceptional phenolic (142.76 mg/g) and flavonoid content (11,432.34 mg/g), further reinforcing its bioactive potential. In vivo findings revealed its ability to reduce MDA levels while enhancing key antioxidant enzymes, suggesting its effectiveness in mitigating oxidative stress. With its significant antioxidant properties and potential health benefits, Mitra tea stands out as a promising natural healthcare solution for antiaging product development, offering a scientifically backed alternative for consumers seeking longevity and wellness.

## 1. Introduction

The free radical theory proposes that an imbalance between antioxidants and reactive oxygen species (ROS) plays a key role in cellular and tissue damage caused by oxidative stress. This condition, characterized by an excess of ROS, is believed to be a major contributor to various diseases, including cancer, cataracts, age-related illnesses, Parkinson's disease, and the aging process itself [1, 2]. Aging is a natural biological process characterized by a gradual decline in physiological functions and increased vulnerability to diseases. According to the World Health Organization (WHO), the global population aged 60 years and older is expected to double from 1 billion in 2020 to 2.1 billion by 2050 [3]. In Asia, where the aging population is growing more rapidly than in any other region, it is estimated that over 25% of the population will be over 60 years old by 2050 [4]. This demographic shift is associated with a rising incidence of age-related diseases such as cardiovascular disorders, neurodegeneration, and metabolic syndromes, all of which have been linked to oxidative stress. Therefore, exploring antioxidant interventions to mitigate aging-related oxidative damage is of increasing scientific and clinical interest.

Recently, there has been growing interest in natural antioxidants from enzymes like catalase (CAT) and superoxide dismutase (SOD), as well as from trace elements and plant sources. The compounds such as ascorbic acid (vitamin C), α-tocopherol (vitamin E), beta-carotene, coenzyme Q10, selenium, zinc, carotenoids, and phenolics are known for their antioxidant properties, effectively neutralizing free radicals [5]. The studies have shown that diets rich in phenolic compounds from plant-based foods can lower the risk of cardiovascular and cerebrovascular diseases and decrease cancer mortality rates [6]. Furthermore, phenolic compounds possess anti-inflammatory properties, making them useful in treating conditions such as skin diseases, rheumatoid arthritis, and inflammatory bowel disease. The presence of antioxidant phytochemicals is associated with a lower risk of mortality from various diseases [7–9]. Recently, there has been a growing interest in natural antioxidants as alternatives to synthetic compounds, especially within alternative and traditional medicine practices globally. Research has focused on various herbs and medicinal remedies from folk medicine to alleviate symptoms, treat diseases, and develop health-promoting supplements, tonic drugs, and longevity medications. For instance, studies on 40 commonly used Chinese medicinal herbs have evaluated the antioxidant activity of different extract fractions. The results suggest that *Radix sanguisorbae, Cortex cinnamomi, Herba taxilli, Semen arecae, Cinnamomum cassia* Presl*, Taxillus sutchuenensis* (Lecomte) Danser, *Areca catechu* Linn., and *Scutellaria baicalensis* Georgi exhibit significant antioxidant activity and/or high total phenolic content (TPC) [10]. *Mitragyna speciosa* (Korth.) Havil., in addition to being used as an antiaging remedy, has also been traditionally employed to alleviate symptoms of malaria and other ailments [11]. In Ayurvedic medicine, the antioxidant properties of herbal remedies have been evaluated using the 2,2-diphenyl-1-picrylhydrazyl (DPPH) assay. At a concentration of 100 μg/mL, *Hemidesmus indicus* demonstrated the highest antioxidant activity (77.0%), followed by *Plumbago zeylanica* (73.41%), *Acorus calamus* (20.88%), and *Holarrhena antidysenterica* (20.06%), highlighting their efficacy in neutralizing free radicals [10]. Additionally, research in Indian Ayurvedic medicine has revealed that *M. speciosa* exhibits various beneficial effects, including antinociceptive, anti-inflammatory, gastrointestinal, antidepressant, antioxidant, and antibacterial properties [12, 13]. *Mitragyna speciosa* (Kratom) has gained growing scientific interest for its diverse pharmacological activities, including antioxidant, anti-inflammatory, and analgesic effects. Traditionally, Kratom has been widely used in Southeast Asia as part of herbal remedies to treat ailments such as colds, diarrhea, and coughs, as well as to promote vitality and overall well-being. In Thailand, *M. speciosa* (Kratom) was classified as a Type 5 narcotic substance under the Narcotic Act B.E. 2522, with legal enforcement beginning in 2001. However, in 2021, Kratom was removed from the narcotics list following the enactment of the Narcotic Act (No. 8), B.E. 2564 [14–16]. This legal reform has led to its reintegration into both modern and traditional Thai medicine. Subsequently, the Department of Thai Traditional and Alternative Medicine (DTTAM), under the Ministry of Public Health, initiated a program to collect and revise classical Thai herbal formulas containing Kratom for further research and development. One such formulation is the *Mitratonic Remedy*, which has been used by traditional Thai healers for over two decades and is recognized for its health-promoting effects. In accordance with the holistic principles of Thai traditional medicine which emphasize multiherb formulations to enhance therapeutic efficacy through synergistic actions, the *Mitratonic Remedy* combines several medicinal plants including Kratom. *Mitragyna speciosa* (Korth.) Havil. is a tropical evergreen tree belonging to the Rubiaceae family, native to Southeast Asia. Commonly known as Kratom in Thailand, Ketum in Malaysia, and Neithum in Laos, its leaves have traditionally been utilized in local communities to alleviate pain, reduce cough and fever, enhance physical endurance, and assist in managing dependence on certain controlled substances [17]. Despite its long-standing traditional use, scientific validation of this formulation remains limited. To bridge this gap, the present study explores the bioactive potential of the *Mitratonic Remedy* in the form of a herbal infusion, herein referred to as Mitra tea. The investigation focuses on its antioxidant properties using both in vitro and in vivo models, aiming to support its development as a scientifically grounded natural healthcare product for future use in wellness and antiaging applications. These formulations can be further developed into natural products for contemporary healthcare applications. Upon reviewing these medicinal formulas, researchers identified numerous traditional remedies with reported medical properties for treating various ailments and promoting well-being. Despite reports from local Thai healers about the effectiveness of these remedies, scientific data to support their claimed benefits remains limited. Based on the known antioxidant properties of polyphenols and flavonoids, we hypothesize that Mitra tea could significantly attenuate oxidative stress, as evidenced by reduced oxidative markers and increased antioxidant enzyme activity, in a methanol-induced oxidative stress model in rats. This lack of scientific evidence is precisely why researchers have focused on developing local herbal formulations further, aiming to unlock their potential for both medical applications and general healthcare. Among these formulations, the Mitratonic remedy has attracted considerable interest due to its use of common local herbs and its traditional role in enhancing vitality and nourishing the body. The Mitratonic remedy consists of seven ingredients: *Mitragyna speciosa* (Korth.) Havil (MS) is used to alleviate pain, reduce cough and fever [17]. *Bridelia ovata* Decne (BO) (local Thai name: Makaa) belongs to the family Euphorbiaceae and the genus Bridelia, which includes over 60 species found across Africa and Asia, especially in Thailand. In traditional Thai medicine, OB is used for various purposes such as relieving pain, balancing bodily elements, reducing fever, treating diarrhea, and promoting wound healing. It is also applied for skin diseases and acts as a diuretic [18]. *Cassia siamea* Lamk. (CS) (local Thai name: Khi-lek) is traditionally used as a mild laxative and sleep-aid [19]. *Cassia alata* (L.) Roxb. (CA) (local Thai name: Chum-het-thet) is a herbal medicine that has been used for treatment of constipation, stomach pain, ringworm, scabies, purities, eczema, herpes, and skin allergy as a laxative in the Thai National List of Essential Drugs [20]. *Fagraea fragrans* Roxb. (FF) (local Thai name: Kan-krao) is leaves and bark for addressing pancreatitis and gastric pains and fever [21]. *Justicia gendarussa* Burm. f. (JG) (local Thai name: Kraduk-kai-dam) is used for bronchitis, inflammations, vaginal discharges, dyspepsia, eye diseases, and fevers [22]. *Azadirachta indica* A. Juss. var. siamensis Valeton (AI) (local Thai name: Sa-daow) is used traditionally for the treatment of inflammation, infections, fever, skin diseases, and dental disorders [23]. These ingredients make the Mitratonic remedy a suitable candidate for further research and development into tea form healthcare products. This study focuses on antioxidant activity evaluated using several in vitro assays including DPPH, 2,2′-azino-bis(3-ethylbenzothiazoline-6-sulfonic acid) (ABTS), ferric reducing antioxidant power (FRAP), metal-chelating activity (MAC), oxygen radical absorbance capacity (ORAC), and nitroblue tetrazolium (NBT), as well as TPC and total flavonoid content (TFC) analysis. Additionally, oxidative stress markers such as malondialdehyde (MDA) and antioxidant enzymes including SOD, CAT, glutathione reductase (GR), and glutathione peroxidase (GPx) were measured in a Wistar rat model to evaluate the antioxidant effects of Mitra tea. Therefore, this study was designed to investigate the antioxidant capabilities of Mitra tea through laboratory experiments and animal models, providing scientific evidence to validate its traditional claims and paving the way for its development as a future healthcare product.

## 2. Materials and Methods

### 2.1. Reagents and Materials

The following instruments and equipment were utilized in this study: UV–visible spectrophotometer (Shimadzu UV-1800, Kyoto, Japan) for absorbance measurement in antioxidant assays; microplate reader (BioTek Synergy HTX, Winooski, VT, USA) for ORAC and NBT assays; centrifuge (Eppendorf 5810R, Hamburg, Germany) for serum and tissue sample preparation; analytical balance (Sartorius Entris224-1S, Göttingen, Germany); water bath (Memmert WNB14, Schwabach, Germany); refrigerated incubator (Binder KB53, Tuttlingen, Germany); homogenizer (IKA T18 digital ULTRA-TURRAX, Staufen, Germany). The reagents used in this study included ABTS, DPPH, Trolox, sodium carbonate, hydrogen peroxide, gallic acid, phosphate-buffered saline (PBS), AAPH, EDTA, Folin–Ciocalteu reagent, ferrous chloride (FeCl_2_), ferric chloride, magnesium sulfate, and nitrotetrazolium blue chloride—all obtained from Sigma-Aldrich (St. Louis, MO, USA). Acetic acid and hydrochloric acid were purchased from J.T. Baker (Phillipsburg, NJ, USA). Additional chemicals including aluminum chloride (AlCl_3_), potassium persulfate (K_2_S_2_O_8_), potassium dihydrogen phosphate, sodium chloride (NaCl), sodium nitrite (NaNO_2_), sodium acetate trihydrate, anhydrous sodium carbonate (Na_2_CO_3_), sodium hydroxide (NaOH), and sodium dihydrogen phosphate were supplied by Ajax Finechem (Taren Point, NSW, Australia). Dimethyl sulfoxide (DMSO) was sourced from Fisher Chemical (Fair Lawn, NJ, USA). TPTZ and ferrozine were obtained from Fluka (Buchs, Switzerland). Solvents such as ethanol and methanol were obtained from Merck (Darmstadt, Germany).

### 2.2. Preparation of Samples

The plant of samples all were purchased from a herbal pharmacy (Triburee Osod-Thai Herbal Store) located in Hat Yai, Songkhla, Thailand. The plant samples were verified and placed at the Applied Thai traditional medicine herbarium of the Department of Applied Thai traditional medicine, School of Medicine, Walailak University, Thailand. The voucher specimen of MS is ATTM No. 66-001; CS is ATTM No. 66-002; BO is ATTM No. 66-003, CA is ATTM No. 66-004, FF is ATTM No. 66-005, JG is ATTM No. 66-006, and AI is ATTM No. 66-007.

To prepare Mitra tea, equal portions (100 g each) of dried leaf powders from seven medicinal plants MS, CS, BO, CA, FF, JG, and AI were thoroughly mixed in a 1:1 ratio. In addition, individual plant extracts were obtained using 100 g of each herb. Both the combined formulation and single extracts were subjected to a conventional decoction process: the plant material was boiled in 1000 mL of distilled water (DI) maintained at 100 ± 2°C for 15 min. Afterward, the mixtures were filtered using Whatman No. 1 filter paper and lyophilized using a freeze dryer (Christ, Germany). The resulting dry extracts were stored at 20°C for subsequent analysis.

### 2.3. Determination of Phenolic Content

The TPC was determined using a modified version of the Folin–Ciocalteu method, as previously reported [24, 25]. In brief, 120 μL of the extract (2.5 mg/mL) was combined with 1 mL of Folin–Ciocalteu reagent and allowed to react for 5 min. Subsequently, 1 mL of 20% (w/v) sodium carbonate was added, and the mixture was vortexed thoroughly. The reaction mixture was then incubated in the dark at ambient temperature for 90 min. Absorbance was recorded at 725 nm. TPC was calculated based on a calibration curve generated using gallic acid (*y* = 0.2432*x* − 0.0064, *R*^2^ = 1), and results were expressed as milligrams of gallic acid equivalents (mg GAE) per gram of extract.

### 2.4. Determination of Flavonoid Content

Flavonoids are plant-derived compounds recognized for their potent antioxidant properties. The TFC was evaluated via the aluminum chloride colorimetric assay, with slight modifications to a previously reported method [24, 26, 27]. In this procedure, 50 μL of the sample extract (2.5 mg/mL) was mixed with 300 μL of 10% (w/v) aluminum chloride, 300 μL of 5% (w/v) sodium nitrite, and 3 mL of DI. The mixture was left to stand at room temperature for 5 min. Subsequently, 2 mL of 1 M sodium hydroxide was added to terminate the reaction, followed by an additional 5-min incubation. Absorbance was measured at 510 nm, and the flavonoid content was calculated and expressed as catechin equivalents per gram of dry sample.

### 2.5. Determination of Free Radical Scavenging Activities by DPPH Radical Scavenging Assay

The DPPH free radical scavenging capacity of the extracts was evaluated following a previously published protocol with slight modifications [28, 29]. Initially, the sample solutions were subjected to twofold serial dilutions to obtain a range of concentrations. A volume of 20 μL from each dilution was transferred into a 96-well microplate, followed by the addition of 20 μL of 0.2 mM DPPH prepared in methanol. The decrease in absorbance at 517 nm was recorded using a microplate reader immediately after mixing and again after a 10-min incubation at room temperature. Methanol served as the negative control, while blanks were prepared by substituting DPPH solution with methanol. The IC_50_ value, representing the concentration required to inhibit 50% of DPPH radicals, was calculated. The percentage inhibition was computed according to equation (1).

(1) $$\begin{array}{c} \text{Scavenging activity}\left(\%\right)=\left[\frac{A_{\text{sample}}-A_{\text{blank}}}{A_{\text{blank}}}\right]\times 100. \end{array}$$

### 2.6. Determination of Free Radical Scavenging Activities by ABTS Radical Scavenging Assay

The ABTS assay was performed based on a previously established protocol [29, 30]. To generate ABTS^+^ radicals, a solution containing 2 mM ABTS and 2.45 mM potassium persulfate was prepared in a 1:1 volume ratio and incubated in the dark at room temperature for 16 h. The resulting solution was then adjusted with ethanol to obtain an absorbance of 0.70 ± 0.05 at 734 nm. A volume of 10 μL of each serially diluted sample (twofold dilution) was added to 1 mL of the ABTS^+^ solution and incubated for 6 min. Absorbance was measured at 734 nm, with Trolox used as the reference standard. The scavenging activity of the extracts was expressed as the IC_50_ value, which represents the concentration required to inhibit 50% of ABTS^+^ radicals (mg/mL). The percentage of ABTS^+^ scavenging activity was calculated according to equation (1).

### 2.7. Determination of Single Electron Transfer–Based FRAP Assay

The ferrous ion-chelating ability of polyherbal extracts was assessed using a colorimetric assay based on a modified version of the FRAP method [31]. The FRAP solution was prepared by combining 10 mL of acetate buffer (300 mM), 1 mL of TPTZ (10 mM), and 10 mL of ferric chloride (20 mM). For the assay, 20 mL of each sample was diluted in ethanol to obtain different concentrations. The diluted samples were added to individual wells of a 96-well microtiter plate and incubated in the dark at 37°C for 30 min. The absorbance, which reflects the formation of a blue complex, was measured at 594 nm. The reducing power was expressed as μM FeSO_4_ per mg of extract.

### 2.8. Determination of MAC

Iron chelation was assessed using a colorimetric assay to measure the ability of antioxidants in the samples to reduce divalent iron (Fe^2+^), following established protocols [32]. Briefly, 0.2 mL of 0.1 mM FeSO_4_ and 0.4 mL of 0.25 mM ferrozine were combined with 0.2 mL of each sample concentration. The mixture was incubated at room temperature for 15 min, and the formation of the ferrous-ferrozine complex was measured by absorbance at 562 nm. EDTA was used as a positive control. The MCA was calculated using equation (2).

(2) $$\begin{array}{c} \text{Metal chelating activity}\left(\%\right)=\left[\frac{A_{\text{sample}}-A_{\text{blank}}}{A_{\text{blank}}}\right]\times 100. \end{array}$$

The absorbance of the control reaction, which does not include the sample, is denoted as A blank, while the absorbance of the reaction containing the plant extract is denoted as A sample.

### 2.9. Hydrogen Atom Transfer–Based Assay and Peroxyl Radical Scavenging Assay

The hydroxyl (OH) radical scavenging activity was measured using the AAPH thermal homolysis method [29, 33]. The assay was conducted in black 96-well plates with PBS (pH 7.4) as the medium. A 25-μL Trolox solution was used as the standard, and samples were tested in 25-μL volumes at various concentrations (2-fold dilutions). The plate was incubated at 37°C, and readings were taken every 5 min for 90 min at excitation 485 nm and emission 535 nm. Antioxidant capacity was expressed as Trolox equivalents per microgram of extract (μM Trolox/μg E), calculated using equations (3) and (4).

(3) $$\begin{array}{c} \text{AUC}=\left(\frac{R_{1}}{R_{1}}\right)+\left(\frac{R_{2}}{R_{1}}\right)+\left(\frac{R_{3}}{R_{1}}\right)+\cdots +\left(\frac{R_{n}}{R_{1}}\right), \end{array}$$

where R1 is the fluorescence reading at the initiation of the reaction and Rn is the final measurement.

(4) $$\begin{array}{c} \text{Net AUC}={\text{AUC}}_{\text{sample}}-{\text{AUC}}_{\text{blank}}. \end{array}$$

### 2.10. Determination of the Superoxide Anion Radical Scavenging Activity [19, 34, 35]

The reaction mixture contained 100 μL of NBT (400 μg/mL) and 0.4 mL of a solution with riboflavin (30 μg/mL), methionine (30 μg/mL), EDTA (20 μg/mL), and plant extract at various concentrations (2-fold dilutions, 4.88–156.25 μg/mL) in 0.05 M PBS (pH 7.4). Superoxide radicals were generated by exposing the mixture to a 20 W fluorescent lamp at 25°C for 25 min. Absorbance was measured at 560 nm, and scavenging activity was quantified as the concentration required to inhibit 50% of the superoxide anion radicals (IC_50_; mg/mL), with Trolox as the reference.

### 2.11. In Vivo Study Design [36, 37]

Male Wistar rats (body weight 200–250 g, age 4 weeks) were obtained from Nomura Siam International. The animals were housed under standard laboratory conditions, including a temperature of 22 ± 2°C, relative humidity of 50 ± 10%, and a 12-h light/dark cycle. Food and DI water were provided ad libitum. Animals in all groups were given free access to food and DI water throughout the experimental period to avoid potential interference from minerals or contaminants that may be present in tap water [38]. After 1 week of acclimatization, the rats were randomly assigned into five groups using a simple randomization method (*n* = 6/group):

  Group 1: Sham, drinking DI water.
  Group 2: Vehicle control, drinking DI water.
  Group 3: High dose, received Mitra tea, a dose of 1000 mg/kg.
  Group 4: Medium dose, received Mitra tea, a dose of 500 mg/kg.
  Group 5: Low dose, received Mitra tea, a dose of 300 mg/kg.

After 14 days, oxidative stress was induced by daily intraperitoneal (IP) injections of methanol at a dose of 2.37 g/kg body weight for 7 consecutive days. The injections targeted the abdominal cavity while carefully avoiding internal organs such as the liver and bladder. A 26-gauge needle was used, with the rat's abdomen gently lifted and its head tilted downward to facilitate safe administration. Test substances were administered concurrently according to the assigned experimental groups. Following the stress induction period, all rats underwent a 16-h fasting period with ad libitum access to water. After fasting, euthanasia was performed via intraperitoneal injection of thiopental sodium (150 mg/kg body weight). A cardiac puncture was then carried out to collect 3–4 mL of blood from the heart. The hypothalamus was extracted by opening the skull, and the adrenal gland, liver, and kidneys were collected through an abdominal incision. Animals were sacrificed on June 11, 2023, for organ collection and further analysis. All the protocols were approved by the Animal Ethics Committee of Walailak University, Thailand (WU-ACUC-66020). The hypothalamus, adrenal gland, liver, and kidney tissues were dissected. One-third of each tissue was fixed in 10% formalin for histopathological analysis using hematoxylin and eosin (H&E) stain. The remaining two-thirds were flash-frozen in liquid nitrogen, then stored at −80°C for the analysis of oxidative markers, including SOD, GR, CAT, ROS, and MDA, in gastrointestinal tissues.

### 2.12. Statistical Analysis

The data are expressed as mean ± standard deviation (*M* ± SD). Each experiment was conducted in triplicate, and statistical analysis was performed using analysis of variance (ANOVA) followed by Duncan's multiple range test (DMRT). Significant differences (*p* < 0.05) among the mean values were determined using SPSS software for Windows Version 19 (IBM Corp., Armonk, NY, USA).

## 3. Results

### 3.1. Determination of Phenolic and Flavonoid Contents

For the Mitra tea sample, TPC was 142.76 ± 5.07 mg GAE per gram of extract, and TFC was 11,432.34 ± 248.98 mg catechin equivalents per gram of extract. Significant differences were observed in the TPC and TFC among the herbal extracts. MS extract exhibited the highest TPC at 215.70 ± 8.54 mg GAE per gram of extract. However, no significant difference in phenolic content was found between MS and BO or AI extracts. Regarding TFC, MS extract also showed the highest content, with a value of 24,246.42 ± 1002.85 mg catechin equivalents per gram of extract. These results suggest that MS extract could be considered the most promising candidate due to its consistently high phenolic and flavonoid contents (Table 1).

### 3.2. Single Electron Transfer–Based FRAP Assay

The ion-chelating effect increased with concentration, showing a significant reducing power. Mitra tea exhibited an FRAP value of 2551.95 ± 80.40 mM FeSO_4_ equivalent/mg sample. This result aligned with the TPC and TFC values. Among the herbal components, AI extract demonstrated the highest FRAP value of 4743.89 ± 66.51 mM FeSO_4_ equivalent/mg sample, although no significant difference was observed compared to the BO extract Table 1.

### 3.3. Determination of MAC

As shown in Table 2, Mitra tea demonstrated a good metal-chelating ability with an IC_50_ value of 1.58 ± 0.26 mg/mL. The metal chelation capacity of CA extract was significantly higher than that of the other herbal components, followed by JG extract with IC_50_ values of 0.22 ± 0.00 mg/mL and 0.62 ± 0.20 mg/mL, respectively.

### 3.4. Determination of Free Radical Scavenging Activities

The antioxidant activity of Mitra tea and its herbal components extract was assessed using two different radical assays: DPPH and ABTS of Mitra tea showed 0.40 ± 0.04 mg/mL, and 0.89 ± 0.02 mg/mL, respectively, in Table 2. Among the herbal component extracts, AI, MS, and FF extracts exhibited significantly stronger radical scavenging activity compared to others, with IC_50_ values of 0.14 ± 0.01, 0.19 ± 0.02, and 0.34 ± 0.05 mg/mL, respectively. The superior radical scavenging ability of the AI extract, demonstrated by both the DPPH and ABTS assays, indicates its strong antioxidative capabilities, which are statistically significant compared to other herbal extracts used in the remedy.

### 3.5. Hydrogen Atom Transfer–Based Assay and Peroxyl Radical Scavenging Assay

The tested extracts scavenged peroxyl radicals in a concentration-dependent manner, as indicated by the inhibition of fluorescence decay. Mitra tea demonstrated significant peroxyl radical scavenging properties, with an ORAC value of 18.505 ± 10.251 μM TE/g extract (Table 3).

### 3.6. Superoxide Anion Radical Scavenging Activity (NBT Assay)

**Figure 1:**
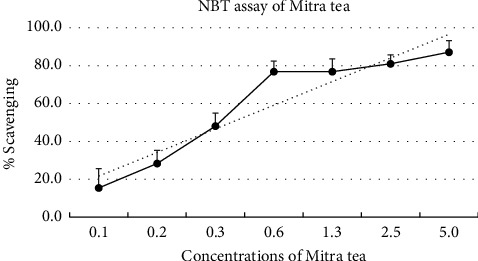
Effects of Mitra tea on percentage of the superoxide anion radical scavenging activity (NBT assay) of different concentrations. All values are presented as the *M* ± SD.

Mitra tea exhibited a dose-dependent scavenging effect on superoxide radicals, with results compared to the known antioxidant Trolox (Figure 1). The IC50 value for superoxide scavenging was 0.19 ± 0.05 mg/mL (Table 3). At a concentration of 5 mg/mL, the inhibition percentage reached 87.10%.

### 3.7. Experimental Animals

**Figure 2:**
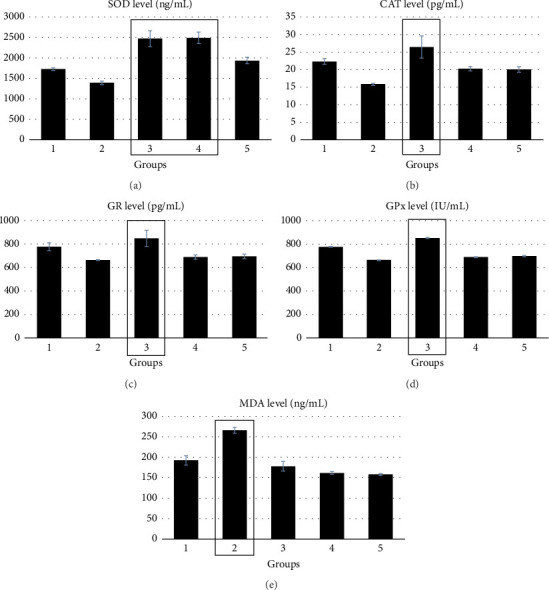
Effects of Mitra tea on SOD (a), CAT (b), GR (c), GPx (d), and MDA (e) in the methanol-induced oxidative stress in gastrointestinal tissue. Groups: (1) Sham, drinking DI water, (2) vehicle control, drinking DI water, (3) high dose, received Mitra tea, a dose of 1000 mg/kg, (4) medium dose, received Mitra tea, a dose of 500 mg/kg, and (5) low dose, received Mitra tea, a dose of 300 mg/kg.

Table 4 and Figure 2 show the levels of MDA, SOD, CAT, GR, and GPx in the gastrointestinal tissues of rats from the five groups. A significant decrease (*p* < 0.05) in MDA levels was observed in the gastrointestinal tissue of Group 5 (low dose of Mitra tea, 300 mg/kg), followed by Group 4 (medium dose, 500 mg/kg) and Group 3 (high dose, 1000 mg/kg) when compared to Group 2 (vehicle control). Additionally, rats in Group 3, receiving the highest dose of Mitra tea, exhibited the highest levels of SOD, CAT, GR, and GPx compared to all other groups.

## 4. Discussion

Aging in mammals is primarily due to natural metabolic and developmental processes, but external sources of free radicals, such as sunlight, smoke, and byproducts of aerobic metabolism, significantly accelerate cellular damage and aging. The free radical theory, proposed in 1956, suggests that exposure to radiation, which generates free radicals, reduces lifespan and induces aging-like changes [29, 39–41]. Antioxidants are essential in slowing the aging process by neutralizing free radicals and preventing cellular damage. These antioxidants can be categorized into enzymatic and nonenzymatic types. Nonenzymatic antioxidants, including vitamins C and E, carotenoids, and plant-derived compounds, prevent free radical chain reactions. Phenolic and flavonoid compounds, known for their antioxidant properties, protect cells from oxidative damage caused by free radicals. These compounds are found in various foods such as fruits, vegetables, and medicinal plants. Examples of these beneficial compounds include gallic acid, epicatechin, quercetin, and flavonoids, which highlight their importance in maintaining health and preventing oxidative stress [42–48].

Phenolic and flavonoid compounds are essential constituents of plants with redox properties that contribute to their antioxidant activity. Previous studies have shown that the antioxidant capacity of natural substances is largely influenced by their phenolic and flavonoid content. In this study, Mitra tea was found to be rich in both phenolic and flavonoid compounds, particularly flavonoids. Similarly, research on Tri-TharnThip tea, a traditional Thai herbal remedy, demonstrated elevated flavonoid concentrations. Detailed analysis of Tri-TharnThip tea identified several flavonoid compounds, which exhibited significant iron-chelating abilities [46, 47]. These findings are consistent with previous studies that highlighted high flavonoid levels and their potential for iron chelation, as confirmed through metal chelation assays. Such results underscore the importance of flavonoids in promoting antioxidant defenses and their role in maintaining health through natural sources.

Iron plays a vital role in physiological functions such as oxygen transport, respiration, and enzymatic processes. However, an excess of iron can lead to oxidative damage in tissues and cells. Chain-breaking antioxidant activity refers to a substance's ability to interrupt the propagation of oxidative reactions by interacting with stable free radicals [48, 49]. This study utilized both metal-chelating and ferric-reducing antioxidant potential assays to evaluate the free radical scavenging properties of the tested samples. The results indicated that Mitra tea effectively functioned as an electron donor, converting free radicals into more stable forms and preventing the propagation of radical chain reactions. Among the herbal components, CA exhibited the most significant MAC, outperforming other samples and supporting findings from previous studies. For instance, leaf extracts of CA demonstrated impressive metal chelation and potent DPPH radical scavenging abilities [46]. These results, together with earlier research, highlight CA as a key herbal agent in Mitra tea for metal chelation. Additionally, GB and MS extracts also exhibited notable metal-chelating properties, comparable to those of CA. In result of this study, Mitra tea exhibited high levels of TPC and TFC, particularly flavonoids, which are known to play a key role in antioxidant defense mechanisms. The strong antioxidant activity observed in our assays is consistent with the high presence of these compounds. These findings align with previous reports indicating that phenolic and flavonoid constituents significantly contribute to the antioxidant potential of herbal teas [50, 51]. The strong antioxidant activity observed in Mitra tea is consistent with its high phenolic and flavonoid contents. This relationship is in line with previous reports demonstrating significant positive correlations between TPC and TFC and antioxidant activities assessed by FRAP, DPPH, and MAC [29]. These findings further support the pivotal role of these compounds in the antioxidant potential of natural products. Therefore, the antioxidant effects observed in Mitra tea may be primarily attributed to its rich phenolic and flavonoid composition.

The antioxidant scavenging activity of Mitra tea and its herbal components was assessed based on their ability to donate hydrogen or scavenge radicals, using the DPPH and ABTS assays. Among all the plants tested for DPPH free radical scavenging, the MS extract exhibited the highest activity and also showed elevated levels of ABTS and DPPH radical scavenging abilities. These results are consistent with previous studies, which demonstrated strong antioxidant capabilities of MS through both DPPH and ABTS assays [52]. Flavonoids were identified as key active compounds in MS, contributing significantly to its antioxidant properties. Notable flavonoids, such as rutin, epicatechin, quercetin, and procyanidin B2, were found to play a major role in its antioxidant effects [52–54].

Mitra tea was further tested for its antioxidant activity against superoxide anions (NBT assay) and peroxyl radicals (ORAC assay), both of which are biologically relevant ROS commonly found in human pathophysiological conditions [54, 55]. The ORAC assay evaluates a substance's capacity to inhibit peroxyl radical–induced oxidative damage, particularly lipid peroxidation [26, 56], while the NBT assay assesses the scavenging of superoxide anions, a major contributor to intracellular oxidative stress [57, 58]. This study is the first to explore the potential of Mitra tea to scavenge free radicals in the body using both NBT and ORAC assays, demonstrating its ability to counteract both superoxide anion and peroxyl radical free radicals. The results demonstrated that Mitra tea exhibited concentration-dependent antioxidant activity in both assays. When compared to other Thai herbal teas, such as Tri-TharnThip (ORAC ≈ 3.11 ± 1.50 μM TE/g extract) [46], Mitra tea showed comparable or even higher scavenging potential, particularly in peroxyl radical suppression. Notably, few studies have assessed both superoxide and peroxyl radical scavenging in herbal formulations rather than isolated compounds. This highlights the novelty of our approach, which employs a traditional Thai multiherb formula. Moreover, the use of biologically relevant radical assays was intended to closely mimic human oxidative stress conditions, thereby enhancing the translational significance of the findings. Given that Mitra tea is being considered for future product development, this comprehensive antioxidant profiling underscores its potential as a functional herbal beverage.

These findings reinforce the free radical theory by demonstrating that polyphenol-rich herbal formulations like Mitra tea can effectively neutralize free radicals, chelate pro-oxidant metals, and thereby reduce oxidative stress in biological systems. By identifying specific herbal components with strong antioxidant mechanisms—such as flavonoid-mediated electron donation and iron chelation—this study not only supports existing concepts of antioxidant defense but also broadens the theory by illustrating the multifaceted roles of plant-derived compounds in mitigating oxidative damage.

The Wistar rat is a strain of white rat that has been widely used in laboratory research since 1906 [59]. It is selectively bred, resulting in consistent physiological responses to drugs and chemical substances. Additionally, its moderate body size, ease of handling, rapid growth, and well-defined life cycle make it ideal for laboratory use. Several of its biological structures are similar to those of humans, particularly the gastrointestinal system, liver, kidneys, metabolic processes, and immune responses. These characteristics make Wistar rats suitable for studies involving pharmaceuticals, herbal medicines, dietary supplements, toxic substances, and more [60, 61]. The sample size of six animals per group was determined based on the minimum requirement set by the Institutional Animal Care and Use Committee (IACUC), which ensures ethical use of animals in accordance with the 3Rs principle (Replacement, Reduction, and Refinement). Additionally, this number is commonly used in prior studies of a similar design to ensure sufficient statistical power. Six rats per group were used, which is commonly accepted as sufficient for detecting moderate to large effects in similar studies [62]. This number also complies with the “resource equation approach”, ensuring an appropriate degree of freedom (E) between 10 and 20 for valid statistical inference. In this study, the total number of animals used in this study was 30, distributed equally into 5 groups with 6 rats each. According to the resource equation approach [63], the degree of freedom is 25. While slightly above the recommended range of 10–20, this sample size was chosen to balance ethical considerations with the need for adequate statistical power to detect significant differences between groups. Methanol was chosen as the oxidative stress inducer in this study due to its well-established role in generating ROS and causing oxidative damage in biological tissues. Methanol metabolism produces formaldehyde and formic acid, which contribute to increased oxidative stress and lipid peroxidation, leading to cellular injury [64]. Previous animal studies have shown that methanol administration elevates oxidative stress markers and induces tissue damage, making it a reliable model for studying oxidative stress–related pathologies [65]. Therefore, methanol is an appropriate agent to induce oxidative stress in this research to evaluate potential protective effects of tested interventions.

In the present study, Wistar rats were selected to evaluate the antioxidant potential of Mitra tea. At a low dose, Mitra tea effectively decreased the levels of MDA, indicating a reduction in oxidative stress, likely due to the tea's antioxidant content. In contrast, higher doses of Mitra tea led to an increase in the activities of antioxidant enzymes such as SOD, CAT, GR, and GPx. While no significant changes in MDA levels were observed at these higher doses, it is important to note that the MDA level in the high-dose group (Group 3) was higher than some treatment groups but remained lower than that of the vehicle control group (Group 2), which received only drinking DI water. This suggests that despite some fluctuations, Mitra tea at a high dose still exerts a protective effect against lipid peroxidation induced by oxidative stress. The observed increase in antioxidant enzyme activities at higher doses may indicate a priming effect, preparing cells for future oxidative stress by enhancing the body's natural defense mechanisms. SOD and CAT, crucial antioxidant enzymes, play a pivotal role in protecting cells from oxidative damage. Interestingly, previous studies have demonstrated that nonsteroidal anti-inflammatory drugs (NSAIDs) can reduce SOD activity in rat gastric tissues, further highlighting the importance of maintaining SOD function in combating oxidative stress [66]. Biological variability, a common feature in in vivo experiments, can contribute to minor inconsistencies in oxidative stress markers such as MDA. Nevertheless, the elevated activities of antioxidant enzymes in the high-dose group support the notion that Mitra tea effectively enhances endogenous antioxidant defenses, underscoring its potential to mitigate oxidative damage despite slight variability in some parameters. CAT, a highly reactive enzyme, is known for its ability to decompose hydrogen peroxide (H_2_O_2_) into water and oxygen, a reaction that helps mitigate cellular damage caused by oxidative stress. Additionally, CAT can also facilitate the production of byproducts such as methanol, ethanol, formic acid, and phenols through hydrogen donation [67].

Mitra tea from Thai folk medicine demonstrates excellent antioxidant activity both in laboratory tubes and in rat experimental. Group 5 (low-dose Mitra tea) showed a reduction in MDA levels, indicating a decrease in oxidative damage. Although no significant changes in MDA levels were observed compared to the high-dose group, the low dose successfully activated antioxidant enzymes (such as SOD, CAT, GR, and GPx). This suggests that Group 5 benefits from effective protection against free radicals. The activation of these enzymes helps prepare cells to better resist oxidative stress in the future. Group 4 (medium-dose Mitra tea) showed a reduction in MDA levels and an increase in the levels of SOD, CAT, GR, and GPx. This indicates a significant activation of antioxidant enzymes, which plays an important role in protecting cells from oxidative damage. Group 3 (high-dose Mitra tea) demonstrated a clear activation of antioxidant enzymes, which contributed to effective protection against oxidative damage. The levels of SOD, CAT, GR, and GPx were the highest in this group, although MDA levels did not decrease significantly. The high activation of these enzymes suggests that the body is better prepared to face oxidative challenges in the future. In summary, Group 3, which received the highest dose of Mitra tea, appears to have the best potential in terms of activating antioxidant enzymes and providing protection against free radicals. Even though MDA levels did not significantly decrease, the elevated levels of antioxidant enzymes indicate effective long-term protection and preparedness against oxidative stress. Thus, Group 3 demonstrated the strongest antioxidant effects and cell protection among the groups.

Although preclinical animal models such as rats are widely used in pharmacokinetic studies, species-specific differences in physiology, metabolism, and hepatic blood flow can limit the translatability of their findings to humans [68]. In this context, interspecies variations particularly in hepatic enzyme expression and xenobiotic biotransformation can lead to discrepancies in pharmacodynamic and toxicological responses [69]. As shown in this study, scaling methods based on individual species—especially monkeys—tended to provide more accurate and less biased predictions of human clearance than rat-based models or purely allometric approaches. Moreover, drug dosages used in rodent models often require careful adjustment through allometric scaling to approximate human-equivalent doses; failure to apply such corrections may result in misleading conclusions [70]. Therefore, while animal models remain essential tools in early drug development, subsequent validation in human-relevant systems is crucial to ensure clinical applicability.

However, this study on Mitra tea is the first time, so there are no reports yet on its other biological activities or its ability to reduce MDA in humans. This is an interesting area for future research, as it could help develop Mitra tea into a natural health-promoting product, for antiaging purposes, and provide another option for healthcare in today's world.

## 5. Conclusions

The current study reveals that Mitra tea demonstrates significantly enhanced antioxidant properties, which may help reduce the levels of free radicals in the body, potentially preventing various diseases and slowing the aging process. These findings suggest that Mitra tea could play a role in health promotion, particularly in counteracting oxidative stress associated with aging and chronic diseases. However, it is important to note that human studies have not yet been conducted to confirm these benefits in real-world applications. As a result, researchers are now focused on advancing Mitra tea into a commercially viable product. This includes the development of Mitra tea blends, which will undergo sensory evaluations to determine consumer preferences, flavor profiles, and overall acceptance. Future research will also aim to further understand the mechanisms by which Mitra tea exerts its antioxidant effects and assess its long-term health benefits. If successful, these efforts could lead to the creation of a health-promoting tea product designed to slow the aging process and improve overall well-being.

## Figures and Tables

**Table 1:** Ferric-reducing antioxidant power (FRAP), total phenolic content, and total flavonoid content of Mitra tea and herbal components.

Sample	FRAP assay	Total contents (mg equivalent/g of extract)	
	(mM FeSO_4_ equivalent/mg sample)	Phenolic compounds	Flavonoids compounds
Mitra tea	2551.95 ± 80.40	142.76 ± 5.07	11,432.34 ± 248.98
MS	2791.09 ± 121.51^b^	215.70 ± 8.54^a^	24,246.42 ± 1002.85^a^
CS	743.41 ± 4.82^c^	35.79 ± 6.46 17^b^	4126.1433 ± 576.65^c^
BO	3696.64 ± 174.17^a^	191.60 ± 2.69^a^	11,253.39 ± 2912.71^b^
CA	824.59 ± 8.79^c^	35.67 ± 8.32^b^	4572.06 ± 275.48^c^
FF	2874.45 ± 18.69^b^	68.60 ± 4.58^b^	14,184.09 ± 241.04^b^
JG	356.05 ± 85.19^d^	38.67 ± 2.42^b^	13,638.43 ± 1633.84^b^
AI	4743.89 ± 66.51^a^	210.81 ± 8.05^a^	11,554.09 ± 703.99^b^

^a–d^Values in the same column (MS-AI samples) with different superscripts are significantly different (*p* < 0.05).

**Table 2:** Effects of Mitra tea and herbal components on metal-chelating activity (MCA) and free radical scavenging capacities.

Sample	MCA assay	Radical scavenging properties (IC_50_; mg/mL)^∗∗^	
	(IC_50_; mg/mL)^∗^	DPPH assay	ABTS assay
Mitra tea	1.58 ± 0.26	0.40 ± 0.04	0.89 ± 0.02
MS	1.54 ± 0.02^b^	0.19 ± 0.02^a^	0.11 ± 0.00^b^
CS	2.67 ± 0.86^d^	1.87 ± 0.20^b^	0.57 ± 0.01^c^
BO	6.99 ± 0.62^e^	4.26 ± 0.11^d^	0.11 ± 0.00^b^
CA	0.22 ± 0.00^a^	1.61 ± 0.11^b^	0.21 ± 0.00^b^
FF	2.31 ± 0.11^d^	0.34 ± 0.05^a^	0.10 ± 0.00^b^
JG	0.62 ± 0.20^a^	3.15 ± 0.38^c^	0.80 ± 0.11^d^
AI	1.89 ± 0.85^c^	0.14 ± 0.01^a^	0.08 ± 0.00^a^
Std.	0.01 ± 0.00	0.03 ± 0.00	0.02 ± 0.00

^∗^IC_50_ of EDTA (a positive control) was 0.01 ± 0.0004 mg/mL.

^∗∗^The IC_50_ values of Trolox obtained from the DPPH and ABTS assays were 0.03 and 0.02 mg/mL, respectively.

^a–e^Values in the same column with different superscripts are significantly different (*p* < 0.05).

**Table 3:** In vitro assay for free radical scavenging capacities of peroxyl radicals and superoxide anions.

Sample	NBT (IC_50_; mg/mL)^∗^	ORAC (μM Trolox/g extract)
Mitra tea	0.19 ± 0.05	18.505 ± 10.251

Abbreviation: ORAC, oxygen radical antioxidant capacity.

^∗^The IC_50_ values of Trolox obtained from the NBT assays were 0.025 mg/mL.

**Table 4:** The activities of MDA, SOD, CAT, GR, and GPx evaluated in the gastrointestinal tissues.

Groups	MDA level (ng/mL)	SOD level (ng/mL)	CAT level (pg/mL)	GR level (pg/mL)	GPx level (IU/mL)
1	192.62 ± 11.63^b^	1730.90 ± 31.59^b^	22.27 ± 0.83^b^	776.01 ± 34.29^b^	55.23 ± 3.89^a^
2	265.98 ± 6.95^#c^	1396.44 ± 43.28^#c^	15.77 ± 0.26^#c^	663.71 ± 6.14^#c^	45.34 ± 0.94^#b^
3	177.97 ± 11.86^∗ab^	2476.55 ± 185.13^∗a^	26.44 ± 3.12^∗a^	850.03 ± 70.68^∗a^	56.68 ± 4.44^∗a^
4	161.90 ± 2.98^∗a^	2490.52 ± 142.03^∗a^	20.27 ± 0.65^b^	688.84 ± 17.45^ab^	45.93 ± 1.16^b^
5	158.31 ± 2.65^∗a^	1935.96 ± 79.76^∗b^	19.97 ± 0.73^b^	695.26 ± 19.45^ab^	46.36 ± 1.30^b^

*Note:* Each measurement was done at least in triplicate, and the values are the mean ± SEM for six rats in each group.

^#^Significant difference compared with Group 1 (control group).

^∗^Significant difference compared with Group 2 (vehicle control group).

^a,b,c^indicate comparisons among Groups 1–5 within each parameter. Values sharing the same letter are not significantly different from each other (*p* > 0.05).

## Data Availability

Data are contained within the article.
